# Caregiver burden in Parkinson’s disease: a mixed-methods study

**DOI:** 10.1186/s12916-023-02933-4

**Published:** 2023-07-10

**Authors:** Angelika D. Geerlings, Willanka M. Kapelle, Charlotte J. Sederel, Emma Tenison, Hilde Wijngaards-Berenbroek, Marjan J. Meinders, Marten Munneke, Yoav Ben-Shlomo, Bastiaan R. Bloem, Sirwan K. L. Darweesh

**Affiliations:** 1 Department of Neurology, Center of Expertise for Parkinson & Movement Disorders, Donders Institute for Brain, Cognition and Behaviour, Radboud University Medical Center, P.O. Box 9101 (Internal Code 914), 6500 HB Nijmegen, The Netherlands; 2 Department of Population Health Sciences, Bristol Medical School, University of Bristol, Bristol, UK; 3 Parkinson Café Nijmegen, Nijmegen, The Netherlands; 4 Scientific Center for Quality of Healthcare, Radboud Institute for Health Sciences, Radboud University Medical Center, Nijmegen, The Netherlands

**Keywords:** Caregiver burden, Parkinson’s disease, Chronic diseases, Mixed methods research

## Abstract

**Background:**

Providing informal care for a person with Parkinson’s disease (PD) can be a demanding process affecting several dimensions of a caregiver’s life and potentially causing caregiver burden. Despite the emerging literature on caregiver burden in people with PD, little is known about the inter-relationship between quantitative and qualitative findings. Filling this knowledge gap will provide a more holistic approach to develop and design innovations aiming at reducing or even preventing caregiver burden. This study aimed to characterize the determinants of caregiver burden among informal caregivers of persons with PD, in order to facilitate the development of tailored interventions that reduce caregiver burden.

**Methods:**

We conducted a cross-sectional study in The Netherlands using a sequential mixed methods approach, entailing a quantitative study of 504 persons with PD and their informal caregivers as well as a qualitative study in a representative subsample of 17 informal caregivers. The quantitative study included a standardized questionnaire of caregiver burden (Zarit Burden Inventory) and patient-related (Beck Depression Inventory, State-Trait Anxiety Inventory, Acceptance of Illness Scale, MDS-Unified Parkinson’s Disease Rating Scale part II on motor functions in daily life, Self-assessment Parkinson’s Disease Disability Score), caregiver-related (Brief Coping Orientation to Problems Experience Inventory, Caregiver Activation Measurement, Multidimensional Scale of Perceived Social Support) and interpersonal determinants (sociodemographic variables including among others gender, age, education, marital status and working status). The qualitative study consisted of semi-structured interviews. Multivariable regression and thematic analysis were used to analyse quantitative and qualitative data, respectively.

**Results:**

A total of 337 caregivers were women (66.9%), and the majority of people with PD were men (*N* = 321, 63.7%). The mean age of persons with PD was 69.9 (standard deviation [SD] 8.1) years, and the mean disease duration was 7.2 (SD 5.2) years. A total of 366 (72.6%) persons with PD had no active employment. The mean age of informal caregivers was 67.5 (SD 9.2) years. Most informal caregivers were female (66.9%), had no active employment (65.9%) and were the spouse of the person with PD (90.7%). The mean Zarit Burden Inventory score was 15.9 (SD 11.7). The quantitative study showed that a lack of active employment of the person affected by PD was associated with a higher caregiver burden. The qualitative study revealed cognitive decline and psychological or emotional deficits of the person with PD as additional patient-related determinants of higher caregiver burden. The following caregiver-related and interpersonal determinants were associated with higher caregiver burden: low social support (quantitative study), concerns about the future (qualitative study), the caregiving-induced requirement of restrictions in everyday life (qualitative study), changes in the relationship with the person with PD (qualitative study) and a problem-focused or avoidant coping style (both studies). Integration of both data strands revealed that qualitative findings expanded quantitative findings by (1) distinguishing between the impact of the relationship with the person with PD and the relationship with others on perceived social support, (2) revealing the impact of non-motor symptoms next to motor symptoms and (3) revealing the following additional factors impacting caregiver burden: concern about the future, perceived restrictions and limitations in performing daily activities due to the disease, and negative feelings and emotional well-being. Qualitative findings were discordant with the quantitative finding demonstrating that problem-focused was associated with a higher caregiver burden. Factor analyses showed three sub-dimensions of the Zarit Burden Inventory: (i) role intensity and resource strain, (2) social restriction and anger and (3) self-criticism. Quantitative analysis showed that avoidant coping was a determinant for all three subscales, whereas problem-solved coping and perceived social support were significant predictors on two subscales, role intensity and resource strain and self-criticism.

**Conclusions:**

The burden experienced by informal caregivers of persons with PD is determined by a complex interplay of patient-related, caregiver-related and interpersonal characteristics. Our study highlights the utility of a mixed-methods approach to unravel the multidimensional burden experienced by informal caregivers of persons with chronic disease. We also offer starting points for the development of a tailored supportive approach for caregivers.

**Supplementary Information:**

The online version contains supplementary material available at 10.1186/s12916-023-02933-4.

## Background

Parkinson’s disease (PD) is the second most common neurodegenerative disorder and currently affects more than 8 million people worldwide [1]. It is characterized by a broad spectrum of motor symptoms, including resting tremor, bradykinesia and postural instability, as well as non-motor symptoms such as cognitive impairment, behavioural dysfunction, sleep disruptions and depression. Given the progressive nature of the disease, people with PD typically experience a progressive impairment in performing daily activities and a similarly progressive loss of autonomy over time, leading to a higher dependency on others in supporting their daily living requirements [2, 3]. Most care is provided by an “informal” caregiver, i.e. a person who does not receive financial compensation for providing care. This is typically a direct family member, such as the spouse or a child of the person with PD [3].

Providing informal care for a person with PD can be a highly demanding process which affects several dimensions of a caregiver’s life [4]. “Caregiver burden” is the extent to which caregivers perceive that caregiving has adverse effects on their emotional, social, financial, physical and spiritual functioning [5]. Previous quantitative research has shown that factors influencing the perceived caregiver burden may be related to the characteristics of the person with PD, with severity of motor impairments and higher dependency on activities of daily living having the greatest impact [6]. In addition, the presence of neuropsychiatric symptoms in a person with PD, such as anxiety, apathy, depression, hallucinations or impaired cognition, significantly increases caregiver burden [7–9]. Caregiver characteristics including low perceived social support, feelings of anxiety and depressive symptoms increase caregiver burden [10, 11]. A qualitative study among informal caregivers of people with PD revealed that mental stress—constant worrying about the person with PD, including their safety—and a perceived loss of the relationship with the person with PD were major contributors to the experience of stress and burden [12].

Previous studies have provided insightful overviews of the factors related to caregiver burden [13, 14], but they lack the integration of both qualitative and quantitative findings which allows us to complement research findings that might be omitted by using only quantitative or qualitative research. Using quantitative surveys followed by qualitative interviews allows us to enrich our understanding of the concept of caregiver burden by allowing individuals to describe their own lived experience in their words rather than to fit their experience into predefined constructs. Filling this knowledge gap will additionally help to develop and design innovations aiming at reducing or even preventing caregiver burden, as the development of innovations is based on the obtained information about the experience of caregivers within the context, the new innovations are carried out.

Notably, higher rates of caregiver burden do not only impact the perceived quality of life of caregivers themselves, but can also have an adverse impact on the quality of care for people with PD they care for [15]. In turn, this negatively impacts the health outcomes of people with PD, thus creating a vicious loop. In addition, informal caregivers play an important role in avoiding or at least delaying the onset of PD-related complications that ultimately necessitate institutionalization of people with PD, thereby allowing people with PD to spend many more years in the community [16], which is one of the core desires of many persons with PD. Therefore, improved insight into the factors that contribute to caregiver burden is necessary to develop tailored interventions to support those informally caring for a person with PD. This study aimed to provide such insight, by applying a mixed methods research design that allows us to compare and contrast both data sets for complementarity as well as additional coverage.

## Methods

### Design

We applied a mixed-method approach using a sequential research design [17], which combines the strengths of quantitative and qualitative approaches. Quantitative research allows us to assess factors impacting caregiver burden in a large and potentially generalizable sample of participants but falls short by trying to fit real-life experiences of people into pre-defined categories. Qualitative research, by contrast, allows people to describe their lived experiences in their own words, but it is limited to a relatively small number of participants whose characteristics do not necessarily represent the source population, which in turn affects the generalizability of the research findings. A mixed methods approach allows us to complement research findings that might be omitted by using only quantitative or qualitative research.

For the purpose of this study, we used the baseline data of people with PD and informal caregivers from a longitudinal research project called PRIME-NL [18]. The PRIME-NL aims to evaluate a new integrated and proactive care management model that is implemented in a certain region within The Netherlands and is compared to usual care [18]. For this purpose, questionnaire data among people with PD, informal caregivers and health care providers is collected over multiple years.

**Fig. 1:**
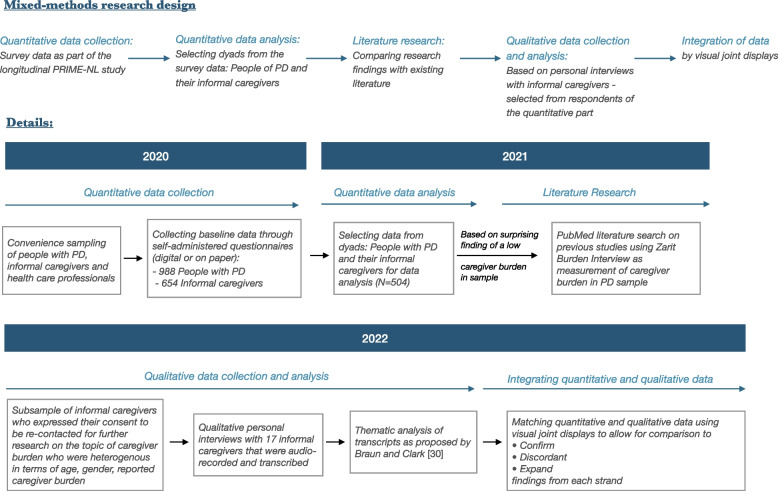
Mixed methods research design. *Next to demographic factors, including for instance age, gender, education and work status

An initial analysis of these data revealed that caregiver burden scores were generally very low in our study population, relative to previously published data and clinical experiences of health care professionals in The Netherlands. In order to find an explanation, we performed a literature research on PubMed to assess whether certain population characteristics could explain the difference. This was not the case. We compared the preliminary results in our study population with previous studies in informal caregivers of people with PD that had also used the Zarit Burden Interview (ZBI) to measure caregiver burden. This search for research-based literature was performed by one author [ADG] in September 2021 using the Medical Subject Heading (MeSH) “Parkinson’s Disease” and relevant keywords including “Burden”, “Caregiver” and “Zarit”. The used PubMed search strategy is presented in Additional file 3: Supplemental file 1. Abstracts and titles were screened to identify relevant studies. Search limits were applied to include only studies reporting on caregiver burden measured by ZBI in a PD population. After the abstract review, full-text articles were assessed for relevance and summarized (Table 1). Therefore, we added personal interviews with informal caregivers to assess whether those would confirm, expand or contradict with our quantitative findings. By using a qualitative approach, participants were able to describe their own lived experiences in their own words instead of trying to fit them into predefined constructs. Through this, we were able to retrieve additional information about the impact of PD and their perceived caregiver burden. The final step consisted of integrating the findings from our quantitative and qualitative studies. Figure 1 displays the applied mixed methods structure for this paper.

**Table 1:** Overview of the literature on caregiver burden in people with PD using ZBI

First author (year)	Country, total (*N*)	Mean ZBI score	Characteristics of caregivers				Characteristics of people with PD		
			**Mean age (SD)**	**Women (%)**	**Spouse (%)**	**Child (%)**	**Mean age (SD)**	**Women**	**Mean disease duration**
Torny (2018) [19]	France, *N* = 38	14.4	67.8 (9.0)	57.9	100	0	70.0 (8.3)	16.0	7.0
Geerlings (2022) [20]	The Netherlands, *N* = 504	15.9	67.6 (9.2)	66.9	90.7	4.2	69.9 (8.1)	36.3	7.2
Santos-Garcia (2015) [21]	Spain, *N* = 121	16.0	60.2 (15.0)	71.9	66.9	30.6	70.9 (8.2)	42.1	6.8
Macchi (2020) [10]	Canada, *N* = 175	17.4	66.1 (11.1)	73.1	n/a	n/a	70.7 (8.1)	29.1	9.5
Yang (2019) [22]	China, *N* = 112	19.6	n/a	58.9	53.6	38.4	n/a	41.1	n/a
Carod-Artal (2013) [23]	Brazil, *N* = 50	20.2	55.7 (13.1)	88.0	78.0	14.0	65.4 (10.3)	20.0	n/a
Rodriguez-Violante (2015) [24]	UK, *N* = 201	21.5	51.6 (13.7)	73.1	n/a	n/a	63.7 (12.6)	46.3	n/a
Martinez-Martin (2015) [7]	Spain, *N* = 562	21.9	59.6 (13.97)	70.5	61.2	29.5	70.8 (9.9)	41.6	8,1
Tan (2020) [25]	Singapore, *N* = 94	23.0	n/a	78.7	46.8	40.5	n/a	36.0	6.9
Trapp (2019) [26]	Mexico, *N* = 95	24.3	51.1 (13.9)	78.0	60.0	23.3	n/a	n/a	n/A
Grün (2016) [27]	Luxembourg, *N* = 59	25.8	63.8 (11.5)	76.3	78.0	0	69.4 (9.8)	30.0	n/a
Martinez-Martin (2007) [11]	Spain, *N* = 80	26.5	61.3 (13.2)	62.0	76.3	18.8	69.4 (11.4)	80 (n/a)	7.7
Hagell (2017) [28]	Sweden, *N* = 66	28.3	69.6 (8.2)	70.8	95.0	2.0	71.5 (7.6)	n/a	9.3
Dotchin (2014) [29]	Tanzania, *N* = 20	30.5	n/a	80.0	n/a	n/a	78.5	n/a	8.0
Klietz (2019) [30]	Germany, *N* = 2019	34.4	70.9 (9.1)	70.8	100	0	74.8 (5.7)	n/a	16.3
Cubo (2014) [31]	Cameroon *N* = 37	35.0	n/a	30.0	n/a	n/a	64.2	n/a	5.8
Miyashita (2006) [32]	Japan, *N* = 646	35.0	64.3 (11.6)	65.0	71.0	21.0	70.4 (9.6)	n/a	8.0
Vatter (2018) [33]	UK, *N* = 136	35.5	69.4 (7.62)	85.3	100	0	73.5 (6.5)	14.7	7.1
Pomponi (2016) [34]	Italy, *N* = 28	42.7	68.6 (6.7)	53.6	100	0	69.5 (5.1)	46.4	8.4
Juneja (2020) [35]	India, *N* = 47.41	47.4	52.3 (6.8)	72.0	76.0	24.0	61.5 (6.7)	32.0	n/a

*ZBI* Zarit Burden Inventory

### Study sample and setting

#### Quantitative study

In the present study, we report results from baseline data of the PRIME-NL study, a prospective observational study among people with PD, informal caregivers and health care professionals in The Netherlands [18]. The study investigates the effect of the novel PRIME Parkinson care model on (I) the health of people with PD, (II) the experience of people with PD and their informal caregivers, (III) the experience of health care professionals involved in the care for people with PD and (IV) the costs of care. A detailed description of the study design has previously been published [18].

In short, the baseline assessment of the study took place between February and December 2020. To be eligible for this study, informal caregivers were required to self-identify themselves as the primary informal caregiver of a person with PD or atypical parkinsonism. “Informal caregiver” was thereby defined as a relative, friend or neighbour who feels involved in the care of a person with PD, for instance, someone who attends medical appointments together with the person with PD, or who is aware of the situation and feels involved. Although a person with PD can have several informal caregivers, the PRIME-NL study strived for the inclusion of one caregiver per person with PD. As the term “caregiver” can have different meanings for different people and can be linked with positive as well as negative associations, potential study participants did not need to define themselves as a caregiver. Actually, some people do not identify as caregivers although they do manage and provide informal care to a person with PD. The inclusion criteria for people with PD were (a) clinical diagnosis of PD or atypical parkinsonism and (b) visited the neurology outpatient clinic of a community hospital at least once during the previous year. Data were gathered through self-administered digital or paper questionnaires. In total, 988 patients and 564 caregivers of people with PD completed the questionnaires. In the PRIME-NL study, there were also cases in which the caregiver participated, but not the person with PD.

We did not perform a sample size calculation for the quantitative analysis in advance, because we assumed that the sample size of the cohort (PRIME-NL) in which the current analyses were embedded would be sufficient to detect meaningful associations. To limit the possibility of a type I error, we performed exploratory factor analysis on the caregiver burden metric in this study (ZBI), which reduced the outcome data from 22 items to 3 factors. This meant that, in our analyses of determinants of caregiver burden, we tested considerably fewer hypotheses than we would have if we had performed no factor analyses. Given the correlation between these factors, and because we had a specific hypothesis based on previous literature for each potential determinant of caregiver we selected, we did not adjust the alpha threshold for multiple-hypothesis testing.

Due to the design of the electronic questionnaire environment, participants were obliged to answer all items of the questionnaires in order to proceed and submit the questionnaire. For the paper-based version of the questionnaire, the PRIME-NL assessment team checked whether all questionnaires were completed, and if in case answers were missing, participants were contacted via telephone to retrieve missing data. Therefore, in the current manuscript, no missing data is reported.

In this current study, we focused on the dyads of persons with PD and their informal caregivers (*N* = 504). We included both people with idiopathic PD as well as atypical parkinsonism. Although people with atypical parkinsonism may experience additional symptoms and signs that are not typically present in PD, such as orthostatic hypotension, early postural instability and faster disease progression, there are still large similarities between the diseases. Next to those similarities in signs and symptoms, the frequently delayed diagnosis of atypical parkinsonism has supported our decision to include caregivers of people with atypical parkinsonism as well in this study. The study was conducted in The Netherlands, where the government encourages informal care to ensure that people are able to live independently (e.g. able to perform basic day-to-day activities such as getting in and out of bed, eating and drinking, getting (un-)dressed and personal hygiene) and participate in social society (e.g. participate in social activities and able to meet other people) take as long as possible [36]. In case people are not able to live independent and participate in society, the assistance of family, friends and neighbours is required which is then defined as providing informal care. Municipalities can help by offering home care services (i.e. receiving additional resources as rental lifting devices or getting assistance for meal services and doing the groceries) or supporting informal caregivers.

The PRIME-NL study was reviewed and approved by the Ethical Board of the Radboud University Medical Center (file number: 2019–5618). All participants provided digital or written informed consent before they took part in the study.

#### Qualitative study

In the baseline caregivers’ questionnaire of the PRIME-NL study, participants were asked if they agreed to be separately contacted for possible participation in additional research on caregiver burden. A total of 289 out of 504 informal caregivers indicated that they were willing to participate in an additional interview study. To prevent over-recruitment and unnecessary extra burden on informal caregivers, and to guarantee a diverse sample, only 48 informal caregivers were pre-selected based on varying ZBI scores and distribution among age and gender. Those 48 informal caregivers received a letter containing information about the study in which they were invited to participate in an interview regarding the impact of PD on their life. We purposefully sampled participants for the interviews based on variation in the distribution of the following characteristics in the total study population: age group, gender, years since diagnosis of PD and caregiver burden scores. In total, seventeen interviews were conducted with informal caregivers. All participants provided written informed consent before the interviews were conducted.

### Data collection and measurement

#### Quantitative study

##### Dependent variable

Caregiver burden was measured by the Zarit Burden Interview (ZBI). ZBI is a validated and most widely used measurement for caregiver burden and has been validated in informal caregivers of people with PD [11, 16]. It covers various domains of caregiver burden, including personal life, social life, interpersonal relationships, health and finances. The scale consists of 22 items which are rated on a 5-point Likert scale ranging from 0 (never) to 4 (nearly always). The ZBI is scored by summing the responses of the individual items (range 0–88). A higher score indicates a higher perceived level of caregiver burden. The severity of the burden was classified into four levels of the total ZBI scores: “little or no burden” (< 21), “mild” (21–40), “moderate” (41–60) and “severe” (> 60) [37].

##### Assessments among informal caregivers

*Brief Coping Orientation to Problems Experienced Inventory (COPE)*. The brief COPE is an abbreviated version of the original 60-item COPE measure which is an inventory of individuals’ coping behaviours [38]. It is a self-reported 28-item scale regarding 14 coping style subscales, with two items assessing each coping strategy. For each statement, participants indicate the extent to which they have been using the different coping strategies on a 4-point Likert-scale ranging from 0 (I usually do not do this) to 3 (I usually do this a lot). Total scores range from 0 to 3 for each coping strategy. The higher the score for a particular coping strategy, the more preferred it is by the participants. The 14 coping styles can further be summarized into three overarching coping styles: (a) problem-focused, (b) emotion-focused and (c) avoidant coping [38].

*Caregiver activation measurement [CG-PAM].* The CG-PAM was used to assess caregivers’ knowledge, skills and confidence for the management of health conditions [39]. It is a validated 13-item instrument with scores ranging from 0 to 100 and a higher score indicating a greater level of activation, which is a positive attribute. Each item is rated on a 4-point Likert scale ranging from 1 (disagree strongly) to 4 (strongly agree).

*Multidimensional scale of perceived social support (MSPSS).* The MSPSS consist of 12 items which can be scored on a 7-point Likert scale ranging from 1 (very strongly disagree) to 7 (very strongly agree) [40]. It has three subscales, each assessed by four items, to assess perceptions of perceived social support from three sources: family, friends and significant others. Overall scores range from 12 to 84 with higher scores indicating greater perceived social support.

##### Assessments among people with PD

*Beck depression inventory (BDI).* The BDI is a 21-item self-reported questionnaire used to measure the severity of depressive symptoms [41]. Each item is composed of four statements, depicting a particular symptom. Participants can score on each item on a 4-point Likert scale ranging from 0 (no symptoms) to 3 (very intense symptoms). The total score indicated minimal, mild, moderate or severe depression.

*State-Trait Anxiety Inventory (STAI).* The STAI is a tool to evaluate anxiety [42] measuring two dimensions: (1) state anxiety, the current emotional state of anxiety, and (2) trait anxiety, the type of anxiety characteristics for the individual’s personality. It is a self-reported questionnaire composed of 40 items and is based on a 4-point Likert scale ranging from 0 (almost never) to 3 (almost always). The total score of each dimension ranges from 20 to 80, and higher scores indicate greater anxiety.

*Acceptance of illness scale (AIS).* The AIS was used to assess to what extent people with PD were able to accept the illness without experiencing negative reactions [43]. The scale consists of 8 items with a 5-point Likert scale ranging from 1 (I strongly agree) to 5 (I strongly disagree). Total score ranges from 8 to 40 with lower scores indicating poorer adaption to the illness.

*Motor symptoms.* To assess the impact of motor symptoms on daily living, part II of the MDS-Unified Parkinson’s Disease Rating Scale (MDS-UDPRS) was used [44]. The scale consists of 13 self-reported items with a 5-point Likert-scale ranging from 0 (normal) to 4 (severe). A higher score therefore indicates greater difficulties with coping with activities of daily living.

*Self-assessment Parkinson’s Disease Disability Score (SPDDS).* The SPDDS is an instrument to assess disabilities in activities of daily living in Parkinson’s disease [45]. The scale consists of 24 self-reported items with a 5-point Likert scale ranging from 1 (able to do alone without difficulties) to 5 (unable to do at all). Total score ranges from 24 to 120 with higher scores indicating more severe impairment.

##### Socio-demographic and other variables

The following sociodemographic data of informal caregivers were assessed and included in this study: gender, age, education, marital status, working status, relationship to people with PD, living situation, years of caregiving and caregiving involvement. In addition, the following sociodemographic data of people with PD or atypical parkinsonism were examined: sex, age, education, marital status, working status, living situation, type of diagnosis and time since diagnosis.

#### Qualitative study

An open-ended interview guide was developed after quantitative analyses of the data set were performed (Additional file 3: Supplemental file 2). The interview guide was divided into three parts, covering different stages of the PD journey: time of diagnosis, current situation and future expectations. The interview guide covered topics raised by the quantitative research findings in greater depth but also allowed flexibility in the interviews so that new themes could emerge. Interviews were conducted by a research assistant [CJS] trained in qualitative research techniques. Although we initially planned to conduct the interviews at the informal caregivers’ house, COVID-19 regulations in The Netherlands at the time of this study made that impossible. Therefore, all interviews were conducted by telephone. Interviews lasted between 30 and 60 min.

### Analysis

#### Quantitative study

The distribution of characteristics of informal caregivers and people with PD was expressed with mean and standard deviation values for continuous variables and percentages for categorical variables. Log transformation was used to handle the right-skewed distribution of ZBI values.

To determine the factors of the ZBI in this study population, we performed an exploratory factor analysis (EFA). Promax rotation was used as the correlation between the factors and was assumed. Kaiser–Meyer–Olkin (KMO) and Bartlett’s test of sphericity were measures used to indicate sampling adequacy. We selected factors with an eigenvalue greater than 1 and by inspecting the scree plot [46]. Items that loaded highly on two factors or had a loading ≤ 0.35 were excluded. The fit of the models was evaluated using the comparative fit index (CFI), and a Cronbach’s alpha value ≥ 0.70 was considered acceptable. Raw scores of ordinal variables were used.

Separate multivariable linear regression models were used to identify predictors of caregiver burden based on knowledge from available literature from which a causal diagram was drawn. In model 1, the following background characteristics of informal caregivers were included in the model: age, gender, marital status, educational level, working status, caregiver involvement, caregiver activation, perceived social support and coping strategies. In model 2, the following background characteristics of people with PD were included: age, gender, educational level, working status, coping strategies, disease duration, depression, anxiety, motor symptoms and activities of daily living. Finally, in model 3, we modelled both characteristics of caregivers and people with PD. We used variance inflation factor (VIF) to detect multicollinearity between the predictors of the model. We found high VIF values (> 5) for gender and age variables of people with PD and informal caregivers indicating a severe correlation. After removing gender and age of people with PD as variables, VIF values were between close to 1 and 3 indicating a moderate correlation. Furthermore, linear regression models were repeated for the factors resulting from the EFA. The level of significance was set at a value of *p* < 0.05. Statistical analyses were carried out using R studio, version 3.6.2.

#### Qualitative study

Contrary to our expectations from clinical observations and existing literature on this topic, a low perceived caregiver burden was reported among included informal caregivers. We therefore wanted to find an explanation by adding in-person interviews. Those interviews allowed us to examine whether the quantitative findings could be confirmed or additional factors impacting caregiver burden identified. All interviews were audio-taped, transcribed verbatim, and entered in Atlas.ti version 9.1.6 for data analysis. For ethical considerations, all identifying information of the participant was removed from the transcripts and pseudonyms were used. Based on the grounded theory methodology, the transcripts were first open-coded to allow for new theoretical possibilities and followed by axial coding to group the codes into a boarder category from which main themes were identified. We followed the six steps of thematic analysis of qualitative research as proposed by Braun and Clark consisting of (1) gaining familiar with the data by reading and re-reading the transcripts, (2) generating initial codes, (3) grouping codes into potential themes, (4) reviewing themes by checking if themes match with the extracted codes, (5) naming the themes and (6) producing the report by finalizing analysis of the selected data extracts [47]. In Additional file 3: Supplemental file 3 the coding tree is displayed. Descriptive statistics were used to summarize the demographic and disease-related variables.

#### Integrating qualitative and quantitative findings

Visual joint displays were used to integrate quantitative and qualitative findings after initial separate analyses [48]. The data are visually brought together to allow comparison for confirmation, discordance or expansion of findings from each strand.

### Validity and reliability

Construct validity was previously examined for the quantitative PRIME-NL study by discussing and testing the questionnaires with an expert panel consisting of patient researchers, informal caregivers, researchers experienced in qualitative health care research and health care professionals. In the PRIME-NL study, we carefully selected instruments that were specific for people with PD and informal caregivers of people with PD. To ensure the trustworthiness of qualitative findings, Guba’s and Lincoln’s criteria were adopted: credibility, transferability, dependability and confirmability [49]. For the qualitative part, the interview guide was developed by researchers [ADG and WMK] and discussed with a former informal caregiver to check for construct validity, i.e. credibility. To enhance transferability, interview participants were recruited from different geographical locations within The Netherlands. To ensure dependability, the coding of the interviews was independently performed by two researchers experienced in qualitative research [ADG and CJS]. Disagreements were resolved during a consensus meeting with other co-authors reached [WMK, SKLD], who were not involved in the analysis process until an agreement was reached. Credibility was ensured by discussing the final codes, themes and interpretation of the content with the research team until an agreement was reached. Confirmability was researched by an expert panel meeting where the findings were presented and discussed with the current and former informal caregivers and researchers with experience and expertise in the area of caregivers of people with PD.

## Results

### Literature search

Table 1 summarizes the results of the research literature on studies that report on the caregiver burden of informal caregivers of PD using ZBI as outcome measurement, including the characteristics of the current study. The reported mean ZBI score ranged between 14.4 and 47.4. Only one other study, conducted in France among only 28 informal caregivers, reported a lower ZBI score than the current manuscript. Based on the comparison between these studies, there is no indication that low perceived caregiver burden can be explained by sample demographics of age and gender.

### Quantitative study

#### Sample description

The characteristics of informal caregivers and people with PD are shown in Table 2. The mean age of informal caregivers was 67.5 years. Most informal caregivers were females, had followed higher education, had no active employment and were the spouse of the person with PD. The majority of cases had PD as their diagnosis, were slightly older, predominantly men, had received higher education and had no active employment.

**Table 2:** Characteristics of informal caregivers and people with PD

	Caregivers of people with PD (*N* = 504)	People with PD (*N* = 504)
**Age, mean + SD**	67.6 + 9.2	69.9 + 8.1
**Women, *n* (%)**	337 (66.9)	183 (36.3)
**Education, *n* (%)**		
Primary education	15 (3.0)	20 (4.0)
Secondary education	139 (27.6)	130 (25.9)
Higher education	350 (69.4)	352 (70.1)
**Marital status, *n* (%)**		
Married	446 (88.5)	434 (86.1)
Living with partner	41 (8.1)	38 (7.5)
Divorced	4 (0.8)	3 (0.6)
Widow/widower	1 (0.2)	15 (3.0)
Single/unmarried	8 (1.6)	10 (2.0)
**Working status, *n* (%)**		
Fulltime employment	42 (8.33)	14 (2.8)
Part-time employment	64 (12.7)	26 (5.2)
Self-employed	34 (6.7)	17 (3.4)
Unemployed	14 (2.8)	3 (0.6)
Retired	332 (65.9)	366 (72.6)
Incapacitated for work	15 (3.0)	71 (14.1)
Others	122 (24.2)	22 (4.4)
**Relationship to people with PD, *n* (%)**		
Partner	457 (90.7)	n/a
Child	21 (4.2)	n/a
Sister or brother	6 (1.2)	n/a
Friend	8 (1.6)	n/a
Others	12 (2.4)	n/a
**Living situation, *n* (%)**		
Living on my own	18 (3.6)	32 (6.3)
Living with my partner	437 (86.7)	432 (85.7)
Living with my partner and children	46 (9.1)	36 (7.1)
Living in an institution, i.e. nursing home	–	2 (0.4)
Living independently, but receive ambulatory support	n/a	1 (0.2)
**Years of caregiving, *n* (%)**		
Less than a year	61 (12.4)	n/a
1 to 5 years	235 (47.9)	n/a
5 to 10 years	89 (18.1)	n/a
More than 10 years	42 (8.6)	n/a
I do not provide care (yet)	64 (13.0)	n/a
**Caregiving involvement, *n* (%)**		
Day and night	129 (25.6)	n/a
During the day	146 (30.0)	n/a
3 to 6 times per week	12 (2.4)	n/a
1 to 2 times per week	19 (3.8)	n/a
Less than once per week	32 (6.3)	n/a
Less than once per month	52 (10.3)	n/a
Very variable	114 (22.6)	n/a
**Caregiver burden, mean + SD**	15.9 + 11.7	n/a
Little or no burden (0–20)	366 (72.6)	n/a
Mild to moderate burden (21–40)	118 (23.4)	n/a
Moderate to severe burden (41–60)	20 (4.0)	n/a
Severe burden (61–88)	–	n/a
**Type of diagnosis, *n* (%)**		
Parkinson’s disease	n/a	485 (96.2)
Atypical parkinsonism	n/a	19 (3.8)
**Time since diagnosis, mean + SD**	n/a	7.2 + 5.2

*PD* Parkinson’s Disease, *n/a* not applicable

#### Zarit burden interview

The mean ZBI total score was 15.9 (SD 11.7), ranging from 0 to 60, with 72.6% of informal caregivers reporting little or no burden, 23.4% mild to moderate burden, 4% moderate to severe and none experienced severe burden category. Cronbach’s alpha was 0.93.

Both the Kaiser–Meyer–Olkin measure of sampling adequacy (*r* = 0.94) and Bartlett test of sphericity measure (5037.1 [degrees of freedom = 231, *p* < 0.0001]) indicated that the sample was appropriate for EFA. EFA yielded three factors with an eigenvalue greater than 1 (Table 3). Two items (7, 15) were excluded because of a loading ≤ 0.35, and two items (11, 19) were excluded because they loaded on two factors. Cronbach’s alpha was ≥ 0.70 for all three factors.

**Table 3:** Exploratory factor analysis

Factors of ZBI	Item loading	Communality^a^	Eigen-value^b^	Per cent of variance	*α*-value
*Factor 1: role intensity and resource strain*			2.97	40.3%	0.90
1. Patient asking for too much help	0.384	0.21			
2. Not enough time for caregiver	0.791	0.53			
3. Worrying about fulfilling different responsibilities	0.597	0.52			
8. Patient is dependent on caregiver	0.691	0.46			
10. Health affected	0.503	0.44			
12. Social life suffering	0.650	0.52			
14. Expected to be the only carer	0.635	0.41			
16. Feel unable to take care of the patient much longer	0.465	0.36			
17. Sense of losing control over life	0.580	0.55			
18. Wish somebody would take care over	0.502	0.42			
22. Feel burdened	0.697	0.60			
*Factor 2: social restrictions and anger*			1.24	7.0%	0.79
4. Embarrassed about patient’s behaviour	0.608	0.27			
5. Feel angry	0.543	0.35			
6. Negative effects on other relationships	0.496	0.26			
9. Feel strained	0.623	0.44			
13. Feeling uncomfortable having friends visit because of patient	0.600				
*Factor 3: self-criticism*			1.13	5.9%	0.76
20. Feel should be doing more	0.818	0.17			
21. Feel could do a better job	0.682	0.24			

Only loadings ≥ .35 are shown

*ZBI* Zarit Burden Interview

^a^Communality = the total amount of variance that can be explained by a given principal component

^b^Eigenvalue = the proportion of each variable’s variance that can be explained by the factors

The first factor included eleven items and represented “role intensity and resource strain” of informal caregivers. The second factor consisted of five items and was labelled “social restrictions and feelings of anger”. The third factor included two items and represented “self-criticism”. The factors accounted for 53.2% of the total variance in answers to the ZBI items. The factors “role intensity and resource strain” (*r* = 0.954, *p* < 0.001) and “social restriction and anger” (*r* = 0.811, *p* < 0.001) correlated strongly with the ZBI total score, and a moderate correlation was found between “self-criticism” and total ZBI score (*r* = 0.563, *p* < 0.001).

#### Multivariable regression for predictors of caregiver burden

Perceived social support (beta = − 0.108, *p* < 0.001, 95% confidence interval (CI): − 0.16 to − 0.06), problem-focus coping (beta = 0.352, *p* < 0.001, 95% CI: 0.21–0.50) and avoidant coping (beta = 0.973, *p* < 0.001, 95% CI: 0.73–1.21) contributed significantly to the explained variance of total ZBI scores (Table 4). Informal caregivers using problem-focus or avoidant coping strategies had a significantly higher perceived caregiver burden, while perceived social support reduced caregivers’ burden. Regarding the characteristics of people with PD working status (beta = − 0.25, *p* < 0.05, 95% CI: − 0.47 to − 0.04), motor impairments (beta = 0.251, *p* > 0.05, 95% CI: 0.04–0.46) and impairment in activities of daily living (beta = − 0.011, *p* < 0.05, 95% CI: − 0.02–0.00) had a significant effect on ZBI total score. In other words, having a partner with PD who is still working and has lower motor impairments reduced the informal caregiver’s burden.

**Table 4:** Characteristics of caregivers and people with PD as predictors for caregiver burden

Predictors	Model I		Model II		Model III	
	*B*	95% CI	*B*	95% CI	*B*	95% CI
***Caregiver assessments***						
*Age*						
< 50 years	Ref	Ref			Ref	Ref
50–70 years	0.067	− 0.28–0.42			0.162	− 0.20–0.52
> 70 years	0.113	− 0.26–0.49			0.160	− 0.23–0.55
Women	0.057	− 0.07–0.19			− 0.014	− 0.16–0.13
*Marital status*						
Married	− 0.001	− 0.22–0.21			0.040	− 0.18–0.26
Unmarried	Ref	Ref			Ref	Ref
*Educational level*						
Primary education	− 0.032	− 0.39–0.33			− 0.064	− 0.45–0.32
Secondary education	− 0.036	− 0.39–0.32			− 0.099	− 0.48–0.28
Tertiary education	Ref	Ref			Ref	Ref
*Work status*						
Working	0.006	− 0.16–0.17			0.060	− 0.11–0.23
Not working	Ref	Ref			Ref	Ref
*Caregiver involvement*						
Day and night	Ref	Ref			Ref	Ref
During the day	− 0.006	− 0.17–0.16			− 0.013	− 0.18–0.16
3 to 6 times per week	0.200	− 0.23–0.63			0.225	− 0.21–0.66
1 to 2 times per week	− 0.232	− 0.59–0.13			− 0.219	− 0.59–0.16
Less than once per week	− 0.232	− 0.51–0.04			− 0.235	− 0.52–0.05
Less than once per month	− 0.085	− 0.31–0.14			− 0.025	− 0.28–0.23
Very variable	0.05	− 0.13–0.23			0.050	− 0.14–0.24
Caregiver activation	− 0.006	− 0.02–0.01			− 0.006	− 0.03–0.01
Perceived social support	− 0.116**	− 0.17 to − 0.07			− 0.108**	− 0.16 to − 0.06
*Coping strategies*						
Problem-focused coping	0.346**	0.20–0.49			0.352**	0.21–0.50
Emotion focus coping	0.200	− 0.13–0.37			0.081	− 0.18–0.34
Avoidant coping	0.947**	0.71–1.19			0.973**	0.73–1.21
**People with PD assessments**						
*Age*						
< 50 years			Ref	Ref	–	–
50–70 years			0.108	− 0.72–0.93	–	–
> 70 years			0.086	− 0.74–0.91	–	–
Women			− 0.003	− 0.17–0.16	–	–
*Educational level*						
Primary education			Ref	Ref	Ref	Ref
Secondary education			− 0.140	− 0.53–0.25	− 0.307	− 0.64–0.03
Tertiary education			− 0.206	− 0.59–0.18	− 0.271	− 0.60–0.06
*Work status*						
Working			− 0.300	− 0.48–0.02	− 0.254*	− 0.47 to − 0.04
Not working			Ref	Ref	Ref	Ref
*Coping strategies*						
Taking action			0.077	− 0.10–0.26	0.066	− 0.09–0.22
Distancing			− 0.117	− 0.27–0.03	− 0.072	− 0.20–0.06
Goal oriented			− 0.040	− 0.20–0.12	− 0.012	− 0.15–0.13
Social support			0.048	− 0.07–0.17	0.004	− 0.10–0.10
Avoidance			0.056	− 0.12–0.23	0.071	− 0.09–0.22
Disease duration			0.004	− 0.1–0.02	0.001	− 0.01–0.01
Depression			0.009	− 0.01–0.02	0.002	− 0.01–0.01
Anxiety			0.005	− 0.01–0.02	0.002	− 0.01–0.02
Motor symptoms			0.145	− 0.10–0.39	0.251*	0.04–0.46
Activities of daily living			− 0.009	− 0.02–0.00	− 0.011*	− 0.02–0.00

Model 1—regression analysis with only caregiver characteristics as predictors. Model 2—regression analysis model with only characteristics of people with PD. Model 3—regression analysis model with both characteristics of caregivers and people with PD: age and gender variables of people with PD were excluded due to severe collinearity with other predictors

^**^ < .001

^*^ < .05

Multivariable linear regression models (Table 5) adjusted for the same covariates showed that avoidant coping behaviour was the only significant predictor for all three burden sub-scales. Furthermore, problem-solved coping, perceived social support and motor impairments were significant predictors for role intensity and resource strain as well as self-criticism. Caregiver activation and impairments in activities of daily living were only significant for self-criticism.

**Table 5:** Multivariate linear regression analysis on total ZBI and subscales*

Dependent variable and significantly associated variables	Coefficient	*p*-value	95% CI
***Dependent variable: total ZBI score***			
Social support	− 0.108	< 0.001	− 0.16 to − 0.06
Problem-focused coping	0.352	< 0.001	0.21–0.50
Avoidant coping	0.973	< 0.001	0.73–1.21
Working*	− 0.254	< 0.05	− 0.47 to − 0.04
Motor symptoms*	0.251	< 0.05	0.04–0.46
Activities of daily living*	− 0.011	< 0.05	− 0.02–0.00
***Dependent variable: role intensity and resource strain***			
Problem-focused coping	0.249	< 0.001	0.13–0.37
Avoidant coping	0.759	< 0.001	0.56–0.96
Social support	− 0.066	< 0.01	− 0.11 to − 0.02
Motor symptoms*	− 0.171	< 0.05	− 0.00–0.34
***Dependent variable: social restriction and anger***			
Avoidant coping	0.770	< 0.001	0.60–0.94
***Dependent variable: self-criticism***			
Problem-focused coping	0.347	< 0.001	0.20–0.49
Avoidant coping	0.624	< 0.001	0.38–0.87
Caregiver activation	− 0.018	< 0.01	− 0.03 to − 0.01
Social support	− 0.058	< 0.05	− 0.11–0.01
Avoidant coping*	0.166	< 0.05	0.01–0.32
Motor symptoms*	0.232	< 0.05	0.02–0.45
Activities of daily living*	− 0.013	< 0.01	− 0.02 to − 0.00

*ZBI* Zarit Burden Interview. Only statistically significant results are displayed in this table

^*^Characteristic of people with PD

### Qualitative study

Interviews were conducted among eleven partners, four children and one friend of a person with PD and one interview with a partner caring for a person with atypical parkinsonism. The characteristics of informal caregivers are displayed in Table 6, together with their ZBI total scores derived from the questionnaire-based data. The mean age of participants was 63.1 (SD 11.4) ranging from 47 to 83 years. Most informal caregivers were women (70.6%) and years of care provision ranged between 1 and 20 years.

**Table 6:** Characteristics of the subsample of participants in the interview study

Nr	Relationship	Disease type	Sex	Age	Working status	Time since PD diagnosis	Duration caregiving involvement	Caregiver burden (ZBI)	
								*Score*	*Category*
C01	Partner	PD	Woman	75	Retired	8 years	> 5 years	48	Moderate to severe burden
C02	Partner	PD	Man	83	Retired	2 years	2–5 years	17	Little or no burden
C03	Partner	PD	Man	70	Retired	1 year	< 1 year	9	Little or no burden
C04	Partner	PD	Man	59	Part time	6 years	2–5 years	30	Mild to moderate burden
C05	Partner	PD	Woman	65	Retired	6 years	> 5 years	11	Little or no burden
C06	Partner	PD	Woman	57	Full time	2 years	1 year	6	Little or no burden
C07	Partner	PD	Woman	58	Part time	9 years	> 5 years	43	Moderate to severe burden
C08	Partner	Atypical parkinsonism	Woman	73	Retired	3 years	< 1 year	13	Little or no burden
C09	Daughter^a^	PD	Woman	50	Part time	2 years	2–5 years	34	Mild to moderate burden
C10	Son	PD	Man	53	Unemployed	2 years	< 2 years	13	Little or no burden
C11	Partner	PD	Woman	47	Full time	5 years	2–5 years	19	Little or no burden
C12	Daughter	PD	Woman	51	Part time	14 years	2–5 years	42	Moderate to severe burden
C13	Partner	PD	Woman	74	Retired	20 years	NA	40	Mild to moderate burden
C14	Partner	PD	Woman	77	Retired	4 years	2–5 years	31	Mild to moderate burden
C15	Son	PD	Man	47	Full time	8 years	1–2 years	5	Little or no burden
C16	Friend	PD	Woman	62	Full time	9 years	2–5 years	15	Little or no burden
C17	Partner	PD	Woman	71	Retired	7 years	NA	11	Little or no burden

*ZBI* Zarit Burden Interview, *NA* missing data

^a^At the time of the interview the mother with PD was deceased

Seven main themes were extracted from the interviews that were related to caregiver burden (Table 7 for illustrative quotes). Notably, the identified themes are not independent concepts but are intertwined. For example, the presence of cognitive decline did not only put a burden on informal caregivers regarding performing additional caregiver duties, including administrative work, but also impacted the flexibility of informal caregivers for their own day planning (theme 3: impact on everyday life) and also the emotional well-being of informal caregivers, as some reported a feeling of guilt when they were mad at their partner with cognitive impairment to not function as they would expect (theme 2).

**Table 7:** Main themes of qualitative analysis and illustrative quotes^a^

Themes			Illustrative quotes
1	Dealing with signs and symptoms of cognitive decline		“I think that cognitive decline is the worst. You cannot really have a conversation anymore.” [C07] “He is actually a big child that you have to take by the hand so that he takes his pills, that he gets dressed, that he goes to the dentist on time.” [C08] “What has become the heaviest challenge is that he has become quite forgetful in recent years. And you know that he cannot do anything about it, it’s the stupid Mr. Parkinson that bothers him, but it can be a huge problem from time to time. Especially, when I am tired myself. Then I sometimes freak out: ‘oh, you have already asked that five times!’ You know that this is not right, it gives you a send of failure that you were that dismissive.” [C14]
2	Psychological and emotional well-being		“I can be cheerful, but my heart is crying. I can easily switch. I can enjoy so many beautiful things, but my life has changes so much that I am always sad. Always. […] I am not jealous of other people. I wish others to be happy. At the same time, I recently looked up old photos from the time we were together […] where we were sitting together under a palm tree and his hands around me. Then I really crave for this memories. [PD] did something to my mental health. The happiness in my life is gone.” [C01]
3	Impact on everyday life		If you live with someone with Parkinson’s Disease, your life is very structured. You cannot longer do something spontaneously. This has all to do with the strict times in which the medications need to be taken. […] I’m fine with it. But it does mean that you live by the hour. By the clock.” [C04] “If you need to stay at home more often, you cancel more things. Things he couldn’t or he didn’t want to. Or if we were going somewhere and he wanted to leave after 10 min. Then your social contacts become less.” [C13]
4	Impact on the relationship	Subtheme: relationship with the person with PD Subtheme: relationship with others	“In the beginning I challenge him. I went beyond my limits by for example cleaning the gutters, so that he would say ‘watch out!’, but he stopped saying it. He is not understanding anymore. He is not seeing it anymore” [C01] “He was a biker. He cycled to Hungary, to Italy, around the Ijsselmeer. He was a very strong and powerful man. When we went bicycling together and we had to wait at a traffic light, he would always say: ‘You’ll get an ice cream soon” and then he would push me like a father would push a child. He would put his hand on my back and push me so that we can sit on a patio immediately. Now it’s completely the other way around. He pushed me, but now I must be in front. I must carry him on my shoulders. That’s now our relationship on all domains. I carry him.” [C01] “He has freezing problems and he falls quite often. But if you see him, you think that it is going well. You do not see that he has Parkinson’s disease. And because of the lack of visible symptoms, many outsiders get a wrong idea of the real impact.” [C13] “When people say ‘if I can ever do something for you’, I do not know what to answer. When someone says to me: “come sit down for a while, I’ll take it over from here’, then it really takes the load off my shoulders. It might be a small difference in nuance, but it does have a big impact […] I really need the initiative from my environment.” [C01]
5	Concerns about the future		“What I find very difficult are the fears that she has about the future. It is not an everyday topic, but it becomes evident now and then. As a young woman she has worked in a nursing home with Parkinson’s patients, and she still has that image of how people then used to be: hanging in a chair or just lying in bed. And she is afraid of that. She can be terribly sad about that […] and I have to deal with her fears.” [C04]
6	Positive impact		“Parkinson taught me to slow down. I never again will say ‘I need to quickly do this’ I do not do two things at the same time, never do anything spontaneously and there are no surprises anymore” [C02] “We are doing it together.” [C11]
7	Coping mechanisms		“When I meditate, I cry. Very often. Then I cry and all is gone.” [C01]

^a^Quotes are translated from Dutch to English

#### Theme 1: Dealing cognitive impairment

Dealing with signs and symptoms of cognitive impairment was reported as the main challenge among the participants and more difficult to accept and to manage compared to motor symptoms. Caregivers described the increasing dependence of the person they care for and the resulting additional tasks they need to provide due to cognitive problems as an everyday challenge. They also expressed their continuous fear of the progression of cognitive impairment and the accompanying loss of the person they used to know. As the person with PD became more dependent and forgetful, participants also felt an increase in negative feelings including anger, sadness, frustration and irritation.

#### Theme 2: Psychological and emotional well-being

Caring for a person with PD was associated with continuous worrying about the health and safety of the person expressed by concerns to leave the person home alone, possible progression of the disease and unforeseeable incidents that would make it impossible for the person with PD to be cared for at home, often causing feelings of anxiety. Moreover, sadness was an often-mentioned emotion, which was on the one hand related to the person with PD as they witnessed the impact of PD on the person as well as feeling unable to help. On the other hand, sadness was also related to participants’ awareness of the loss they experienced in terms of relationship with the person with PD and to how it restricted them in their own daily living. Participants also felt guilty as they believed that they were not doing enough for the person with PD or they were not being patient enough in case of cognitive impairment.

#### Theme 3: Impact on everyday life

Living with a person with PD was often associated with a change in routines and perceived restrictions on everyday life. Participants reported that they felt strained in their freedom and had to deal with unmet needs, including having less time for leisure activities that they previously enjoyed (i.e. together going out for dinner, travelling). Planning activities outside the daily structure, such as visiting the family or friends, posed a real challenge considering the medication intake and current physical and mental health of the person with PD. Some participants indicated that this consequently led to a loss of social contacts, which increased by not being able to leave the person with PD alone at home due to safety risks.

#### Theme 4: Impact on the relationship

##### Relationship with the person with PD

As the person with PD lost their functional and/or cognitive abilities, it becomes increasingly difficult for the person to take initiative and action resulting in that certain tasks were left behind, not completed or that they needed to support the person with PD with activities that they could not do independently, i.e. cooking and eating. Although the informal caregivers expressed understanding for the inability to act, they also described that they felt unheard and unseen for the additional tasks they were doing. In addition, participants reported that they realized how their life had changed in a way that they felt to be more emotionally disconnected to the person with PD. This was in particular the case of cognitive decline, and behaviour was compared to that of a child instead of a partner. Participants felt to have lost the person they have been knowing, mostly due to the role reversal of becoming a carer instead of being the partner anymore. Moreover, participants reported that PD negatively impacts sexual intimacy among spouses due to the impact of motor and non-motor symptoms as well as decreased sexual interest caused by being in the role of caregiver for the person with PD.

##### Relationship with others

Participants mentioned that PD had an impact on their relationship with others, both family members as well as people outside family life. As mentioned above, they reported that they had partly given up their social life due to the lack of flexibility and independence. Moreover, participants felt confronted with incomprehension from the environment as the signs and symptoms of PD are often invisible to outsiders and consequently, also the burden of caring.

#### Theme 5: Concerns about the future

Uncertainty about the future was another major concern and perceived stressor for participants. On the one hand, they needed to deal with their own concerns about the future, especially with the fear of losing more autonomy over their life and to not be able to keep caring for the person. On the other hand, they also needed to deal with the fear around the unknown progression of the person with PD and future impact of the disease.

#### Theme 6: Positive impact

Although caring for a person with PD was associated with stressors, some participants could also think about the positive aspects of PD. One recurring named benefit was the calmness that resulted from living a more structured life. In this sense, the caregivers had adapted positively to the changed situation. Another benefit participants experienced was to be more connected to the person with PD, leading to a stronger relationship and the relieved feeling that they did not need to deal with the disease and the impact on their own life.

#### Theme 7: Coping mechanisms

While some participants reported emotional coping through crying as the most often expressed way to reduce feelings of stress and thoughts of fear, others gained strength from taking time for themselves (i.e. being a weekend away without the person with PD) or by engaging in regularly social activities (i.e. doing yoga or other sports or hiking). Others also reported that being prepared and thinking about possible future scenarios helped to cope with the stress (i.e. being forced to move or to renovate the house to meet the needs of the person with PD).

### Integrating qualitative and quantitative findings

Integrating the quantitative and qualitative research findings revealed partially overlapping as well as complementary results. The quantitative results showed that a higher perceived social contact was associated with a lower perceived caregiver burden. The qualitative results expand this finding by distinguishing between two relationships: relationship with the person with PD and relationship with others. In addition, quantitative findings revealed that informal caregivers might also be confronted with incomprehension due to the invisibility of the disease which negatively impacts perceived caregiver burden. With regard to coping mechanisms, the quantitative findings demonstrated that problem-focused and avoidant coping styles were associated with a higher caregiver burden. Qualitative findings were discordant, as planning for the future was perceived as being prepared and lowering perceived caregiver burden. Moreover, the qualitative findings expanded the quantitative findings by showing that informal caregivers used emotional coping which was also associated with a lower perceived caregiver burden. Quantitative findings revealed that informal caregivers were more burdened when the person with PD reported higher motor impairments. Qualitative findings confirmed this result and expanded on the impact of non-motor symptoms, especially in the case of cognitive decline. Finally, qualitative analysis expanded the quantitative findings by revealing four additional themes that were related to a lower perceived caregiver burden: concern about the future, perceived restrictions and limitations in performing daily activities due to the disease, and negative feelings and emotional well-being. Finally, qualitative results also revealed that PD can be experienced in some positive ways with regard to living a structured life and having a stronger relationship with the person with PD which was not captured by the quantitative analysis. Persons with PD report similar positive experiences that come with being diagnosed with PD, known as “silver linings” [50]. Table 8 provides an overview of the factors that are associated with caregiver burden based on the integrated data analysis.

**Table 8:** Merged data from quantitative and qualitative research findings

Topic	Quantitative findings	Qualitative findings	Integrated findings
Relationships and social support	Informal caregivers that reported more perceived social support, reported a lower perceived caregiver burden	Informal caregivers experienced several challenges regarding the *relationship with the person with PD* that impacted the perceived caregiver burden. Several informal caregivers reported role-reversal, sense of loss of the relationship, feeling disconnected from person with PD and becoming more and more a carer than a partner. By contrast, others reported that PD has deepened the relationship with the person with PD and increased mutual understanding Informal caregivers also reported that PD also impacted their *relationship with others*. They felt confronted with incomprehension from their surroundings and also felt a lack of flexibility and independence to continue with their social life	**Expansion:** PD impacts the relationship between the informal caregiver and the person with PD, which can have negative as well as positive impact on perceived caregiver burden **Confirmation:** Social support has a positive impact on perceived caregiver burden
Coping strategies	Problem-focus and avoidant coping behaviour was associated with a higher perceived caregiver burden	Informal caregivers reported the use of emotional coping as well as problem-focused to be prepared for the future	**Discordance:** Problem-focused coping can have a positive as well as negative impact on perceived caregiver burden
Motor and non-motor symptoms	Informal caregivers reporting that the person with PD had higher motor impairments and higher impairments in activities of daily living and had a higher perceived caregiver burden	Informal caregivers reported that the burden of dealing with signs and symptoms of cognitive decline outsight the impact of motor symptoms. Cognitive decline put extra burden on informal caregivers through a higher dependence of the person with PD on support from the informal caregiver, including performing additional tasks. In addition, it was related to the fear of losing the person they were used to know, and the forgetfulness may lead to negative feelings such as sadness, anger and frustration	**Expansion:** Severity of motor symptoms and the related impairment of performing activities of daily living negatively impact perceived caregiver burden Informal caregivers reported that signs and symptoms of cognitive decline have a more severe impact on perceived caregiver burden
Concerns about the future	–	Informal caregiver indicated that they were confronted with both: (1) fear and worries of the person with PD, related to disease progression and severity of symptoms, and (2) own fears and worries about the future way of living and dealing with the impact of PD	**Expansion:** Concerns of the impact of PD on one’s own future as well as on the disease progression and coping of the person with PD negatively impacts perceived caregiver burden
Impact on everyday life	–	Informal caregivers reported perceived restrictions and limitations due to PD, which included a lack of freedom and a lack of autonomy due to a change in daily routines	**Expansion:** Lack of freedom and autonomy of informal caregivers negatively impacts perceived caregiver burden
Psychological and emotional well-being	No association was found for the impact of feelings of anxiety and symptoms of depression of the person with PD on perceived caregiver burden	Informal caregivers worried about the disease progression, as well as the concern to not be able to leave the person with PD home alone. In addition, informal caregivers reported a feeling of sadness as they experience a sense of losing the relationship, feeling to live a different life and the need to give up social and family life. Moreover, informal caregivers reported feeling guilty when they felt unable to deal with cognitive and behavioural impairments	**Expansion:** Informal caregivers’ emotion and psychological well-being impact perceived caregiver burden
Positive impact of PD	-	Informal caregivers reported that PD also had a positive impact on their life and relationship. They experienced more calmness due to living a more structured life and a stronger relationship with the person with PD	**Expansion:** PD has not only a negative impact on the life and relationship of informal caregivers, which positively impacts the caregiver burden

## Discussion

By using a sequential mixed methods research approach with both qualitative and quantitative data, this study has shed new light on the impact of PD on informal caregivers and identified factors affecting the perceived caregiver burden. Taken together, our findings indicate that living with or providing care for a person with PD can pose considerable emotional, psychological and social challenges on informal caregivers. While some experiences are fairly more commonly shared, others remain very unique to each informal caregiver’s individual journey and their own perceived impact of motor and non-motor symptoms.

In the qualitative part of our study, a change in the interpersonal relationship between the informal caregiver and the person with PD was a determinant of the perceived caregiver burden. Specifically, caregivers reported experiencing changing power and role dynamics, including a sense of loss of the relationship that they used to have, and transition from an equal partner to that of an adult–child. This corroborates previous findings in which informal caregivers reported that they did not feel as partners anymore given the inequality in the relationship [51] but also adds the impact of PD onto relations with others. In line with previous research [52], our study revealed that not knowing how and when the disease will progress is emotionally taxing for both people with PD and their informal caregivers. Informal caregivers expressed uncertainty with regard to disease progression and daily functioning, including the risk of falls and not being able to stay safely alone at home due to severe motor and non-motor symptoms. However, our study also revealed uncertainties among informal caregivers regarding their own future life, a topic that is much less commonly studied in the PD field. Feeling the need to stay at home and take care of the person with PD was also related to a loss of freedom, sense of isolation and loss of social life, factors that have also been reported in previous studies [53–55]. The qualitative research also highlighted that cognitive decline was reported as one of the most important factors contributing to perceived caregiver burden, a topic not covered by the quantitative part of our study.

The quantitative analysis revealed that informal caregivers who received more social support from their environment experienced lower perceived caregiver burden. In addition, the analysis revealed that those informal caregivers using an avoidant or problem-focused coping style reported a higher caregiver burden. Previous research found that negative coping styles are linked to negative emotions, such as worry and fear, leading to more mental and physical stress [56]. In contrast, positive coping styles such as problem-focus coping appeared to be protective against the development of burden [16, 57]. It is likely that cultural aspects play a role in the observed difference. Culture-specific beliefs and contexts might play a crucial role in how people appraise and respond to stressful life situations [58]. It can be hypothesized that in a culture where caring for a person with a chronic disease is more commonly shared among family members, seeking social support might be more common as a chosen coping strategy, which is known to be protective against caregiver burden.

Another relevant finding is that caregiving is not only associated with negative impacts. Positive outcomes such as a stronger relationship with the person with PD and living a more structured life were mentioned as positive outcomes of the impact of PD. Few studies have previously addressed the positive aspects of caregiving among people with PD and Alzheimer’s disease [23, 50, 59].

The identified factors impacting caregiver burden of informal caregivers of people with PD are not new in the broader field of scientific literature on caregiver burden in chronic diseases, including, but not limited to PD [4, 60]. Whereas some disease-specific factors such as signs and symptoms of cognitive decline reduce the generalizability of our study findings to the field of neuro-degenerative disease, others are also commonly identified among other chronic diseases.

### Strengths and limitations

Previous research about caregiver burden in PD reported only either quantitative or qualitative results, but the interplay had thus far not been studied. Through the combination of both methodologies, we generated a richer and more comprehensive picture of caregiver burden, which would have not been possible using only one research method. Quantitative analyses allowed us to include an extensive consideration of which characteristics of both, people with PD and informal caregivers, impact the perceived caregiver burden. In contrast, the qualitative interviews allowed us to explore the in-depth experience of the impact of PD from the perspective of the informal caregivers. In addition, the study included a large sample of dyads of people with PD and their primary informal caregiver allowing to explore the impact of both disease-related and caregiver-related characteristics on the caregiver situation and the multidimensional concept of caregiver burden. Compared to previous observational studies in Parkinson’s disease using data from people with PD and informal caregivers, our study had the largest sample size providing more power to the robustness of findings. In our qualitative part, we conducted 17 interviews with informal caregivers of PD and reached data saturation as no insights were derived from the interviews which made additional interviews unnecessary.

However, the study is not without limitations. First, we only included dyads of people with PD and their informal caregivers. As we are not aware of the reasons for the nonparticipation of people with PD when only the person with PD participates, we cannot rule out that our sample selection led to the exclusion of informal caregivers associated with a person with PD who is not capable to participate in the study due to disease progression and severity. This clearly impacts the generalizability of our study findings. In addition, there is a potential for sampling bias, as those who participated in the study were motivated to do so. According to the average ZBI, our sample has a disproportionate number of mildly burdened informal caregivers (2nd lowest mean score out of 20 studies—see Table 1) and only 4% of the included respondents reported moderate to severe burden. This small sample of burdened informal caregivers impacts the generalization of the results to the broader Dutch population with PD. Several reasons might explain the observed low caregiver burden in our study sample. First, it might be that informal caregivers who would score as moderately or even highly burdened are underrepresented in this study. It is reasonable that these more burdened individuals do not have time to participate in research studies and are more likely to decline study invitations and to use the time and energy for caregiver responsibilities. In line with this point, our sample of people with PD is younger compared to the broader Dutch PD population [61]. It is conceivable that caring for an older person with PD impacts the perceived caregiver burden due a higher dependence of the person with PD on assistance in daily life activities and chance of comorbidities that increase with age. In addition, it cannot be ruled out that several informal caregivers are involved in providing care to the person with PD and the sharing of responsibilities might lead to a lower perceived strain among informal caregivers.

The current study focused on baseline data of the PRIME-NL data. Serial follow-up measurements are planned in this cohort, which means that future investigations on this cohort could assess the change in caregiver situation over time and across caregiver trajectories. Finally, the study focused only on informal caregivers living in The Netherlands. It is likely that cultural aspects and the organization of the health care system, including for instance reimbursement of health care costs, impact the perceived caregiver burden. A cross-cultural study design would allow us to examine what insights can be generalized and what are due to cultural differences. Future work will compare these findings to the sister PRIME-UK study [62] which has collected similar data to see whether there are Dutch and English differences in reported carer burden after adjusting for socio-demographic and clinical differences between the samples.

### Implications for future research

Future research on this topic should focus on further determining factors to measure caregiver burden in a personalized manner. As our study provides baseline data for longitudinal follow-up, future studies embedded in this cohort will be able to assess the change in caregiver situation over time and across caregiver trajectories. Furthermore, aside from future studies within this cohort, cross-disease research will provide important insights into whether the factors we identified are PD-specific or rather relevant across different diseases. Additionally, young age at disease onset (e.g., < 50 years) should be explored as a factor that could affect caregiver burden, given the considerable impact of PD on daily life when an individual is diagnosed with PD at a young age. Moreover, future research should focus on exploring factors that could impact caregiver burden in which diversity plays a key role, such as cultural aspects, health literacy and gender.

### Implications for clinical practice

The ZBI is one of the most widely used instruments for assessing caregiver burden. In clinical practice, the assessment of burden among informal caregivers is important for identifying those that are at risk and—if appropriate—to instal a timely intervention. However, to be successful, interventions must be tailored to the individual needs and preferences of the informal caregiver, because what is perceived as a burden and which factors contribute to this will vary between individuals. As such, it is important to use instruments that measure aspects of burden that are relevant for the population under study. Findings from qualitative studies could serve as a basis for a PD-specific version of the ZBI instrument, by adding items that cover contextual and disease-specific factors that impact perceived caregiver burden. Our study, for instance, revealed the impact of cognitive decline on the perceived caregiver burden, which is not covered by the ZBI as it is a general and not disease-specific instrument. Next to considering the individual experience of burden, our findings also emphasize the need to take the coping styles of the informal caregivers into account in order to provide personalized care that strengthens the self-management skills of the caregivers. Finally, perceived social support can help to reduce or even prevent the development of perceived caregiver burden, leaving important chances for enhancing the social network of the informal caregivers, or to stimulate peer-to-peer support [20].

## Conclusion

The burden experienced by informal caregivers of persons with PD is determined by a complex interplay of patient-related, caregiver-related and interpersonal characteristics. It can have adverse effects on the caregiver’s emotional, social, financial, physical and spiritual functioning. At the same time, caring for a person with PD is not always only associated with negative consequences but can also have a positive impact on the relationship. Understanding caregivers’ unique experiences of caring for a person with PD allows for the opportunity to better support the caregiver by adequately responding to the caregiver’s needs and wishes.

## Supplementary Information

**Additional file 1: Fig. S1.** Mixed methods research design. **Fig. S2.** Conceptual model.

**Additional file 2: Table S1.** Overview of the literature on caregiver burden in people with PD using ZBI. **Table S2.** Characteristics of informal caregivers and people with PD. **Table S3.** Exploratory factor analysis. **Table S4.** Characteristics of caregivers and people with PD as predictors of caregiver burden. **Table S5.** Multivariate linear regression analysis on total ZBI and subscales. **Table S6.** Characteristics of subsample of participants in interview study. **Table S7.** Main themes of qualitative analysis and illustrative quotes. Table 8 – Merged data from quantitative and qualitative research findings.

**Additional file 3: Supplemental file 1.** PubMed search strategy. **Supplemental file 2.** Translated interview guide. **Supplemental file 3.** Code tree.

## Data Availability

The data generated during the present study are available from the corresponding author upon reasonable request.

## Abbreviations

AIS: Acceptance of Illness Scale
BDI: Beck Depression Inventory
CFI: Comparative fit index
CG-PAM: Caregiver Activation Measurement
CI: Confidence Interval
Cope: Brief Coping Orientation to Problems Experience Inventory
EFA: Exploratory factor analysis
KMO: Kaiser-Meyer-Olkin
MDS-UDPRS: MDS-Unified Parkinson’s Disease Rating Scale
MeSH: Medical Subject Heading
MSPSS: Multidimensional Scale of Perceived Social Support
PD: Parkinson’s disease
SD: Standard deviation
SPDDS: Self-assessment Parkinson’s Disease Disability Score
STAI: State-Trait Anxiety Inventory
VIF: Variance Inflation Factor
ZBI: Zarit Burden Inventory
