# Supplementary material for: Caregiver burden in Parkinson’s disease: a mixed-methods study
Source: BMC Med. 2023 Jul 10;21:247. doi: 10.1186/s12916-023-02933-4 (PMC10332089; doi:10.1186/s12916-023-02933-4)
Supplement: Supplementary file 1 — Additional file 1: Fig. S1. Mixed methods research design. Fig. S2. Conceptual model. [file 12916_2023_2933_MOESM1_ESM.docx]

** Figure 1: Mixed methods research design**

**Figure 2: Conceptual mode**
