# Supplementary material for: Caregiver burden in Parkinson’s disease: a mixed-methods study
Source: BMC Med. 2023 Jul 10;21:247. doi: 10.1186/s12916-023-02933-4 (PMC10332089; doi:10.1186/s12916-023-02933-4)
Supplement: Supplementary file 2 — Additional file 2: Table S1. Overview of the literature on caregiver burden in people with PD using ZBI. Table S2. Characteristics of informal caregivers and people with PD. Table S3. Exploratory factor analysis. Table S4. Characteristics of caregivers and people with PD as predictors of caregiver burden. Table S5. Multivariate linear regression analysis on total ZBI and subscales. Table S6. Characteristics of subsample of participants in interview study. Table S7. Main themes of qualitative analysis and illustrative quotes. Table 8 – Merged data from quantitative and qualitative research findings. [file 12916_2023_2933_MOESM2_ESM.docx]

| **Table 1: Overview of literature on caregiver burden in people with PD using ZBI*** | | | | | | | | | | | | | |
| --- | --- | --- | --- | --- | --- | --- | --- | --- | --- | --- | --- | --- | --- |
| **First author (year)** | **Country, Total (N)** | **Mean  ZBI score** | **Characteristics of caregivers** | | | | |  | **Characteristics of people with PD** | | | | |
|  |  |  | **Mean age (SD)** | | **Women (%)** | **Spouse (%)** | **Child (%)** |  | **Mean age (SD)** | | **Women** | **Mean disease duration** | |
| Torny (2018) [48] | France, N= 38 | 14.4 | 67.8 (9.0) | | 57.9 | 100 | 0 |  | 70.0 (8.3) | | 16.0 | 7.0 | |
| Geerlings (2022) | The Netherlands, N=504 | 15.9 | 67.6 (9.2) | | 66.9 | 90.7 | 4.2 |  | 69.9 (8.1) | | 36.3 | 7.2 | |
| Santos-Garcia (2015) [49] | Spain, N=121 | 16.0 | 60.2 (15.0) | | 71.9 | 66.9 | 30.6 |  | 70.9 (8.2) | | 42.1 | 6.8 | |
| Macchi (2020) [50] | Canada, N=175 | 17.4 | 66.1 (11.1) | | 73.1 | n/a | n/a |  | 70.7 (8.1) | | 29.1 | 9.5 | |
| Yang (2019) [51] | China, N=112 | 19.6 | n/a | | 58.9 | 53.6 | 38.4 |  | n/a | | 41.1 | n/a | |
| Carod-Artal (2013) [43] | Brazil, N=50 | 20.2 | 55.7 (13.1) | | 88.0 | 78.0 | 14.0 |  | 65.4 (10.3) | | 20.0 | n/a | |
| Rodriguez-Violante (2015) [52] | United Kingdom, N=201 | 21.5 | 51.6 (13.7) | | 73.1 | n/a | n/a |  | 63.7 (12.6) | | 46.3 | n/a | |
| Martinez-Martin (2015) [7] | Spain, N=562 | 21.9 | 59.6 (13.97) | | 70.5 | 61.2 | 29.5 |  | 70.8 (9.9) | | 41.6 | 8,1 | |
| Tan (2020) [53] | Singapore, N=94 | 23.0 | n/a | | 78.7 | 46.8 | 40.5 |  | n/a | | 36.0 | 6.9 | |
| Trapp (2019) [54] | Mexico, N=95 | 24.3 | 51.1 (13.9) | | 78.0 | 60.0 | 23.3 |  | n/a | | n/a | n/A | |
| Grün (2016) [55] | Luxembourg, N=59 | 25.8 | 63.8 (11.5) | | 76.3 | 78.0 | 0 |  | 69.4 (9.8) | | 30.0 | n/a | |
| Martinez-Martin (2007) [11] | Spain, N=80 | 26.5 | 61.3 (13.2) | | 62.0 | 76.3 | 18.8 |  | 69.4 (11.4) | | 80 (n/a) | 7.7 | |
| Hagell (2017) [56] | Sweden, N=66 | 28.3 | 69.6 (8.2) | | 70.8 | 95.0 | 2.0 |  | 71.5 (7.6) | | n/a | 9.3 | |
| Dotchin (2014) [57] | Tanzania, N=20 | 30.5 | n/a | | 80.0 | n/a | n/a |  | 78.5 | | n/a | 8.0 | |
| Klietz (2019) [58] | Germany, N=2019 | 34.4 | 70.9 (9.1) | | 70.8 | 100 | 0 |  | 74.8 (5.7) | | n/a | 16.3 | |
| Cubo (2014) [59] | Cameroon N=37 | 35.0 | n/a | | 30.0 | n/a | n/a |  | 64.2 | | n/a | 5.8 | |
| Miyashita (2006) [60] | Japan, N=646 | 35.0 | 64.3 (11.6) | | 65.0 | 71.0 | 21.0 |  | 70.4 (9.6) | | n/a | 8.0 | |
| Vatter (2018) [61] | United Kingdom, N=136 | 35.5 | 69.4 (7.62) | | 85.3 | 100 | 0 |  | 73.5 (6.5) | | 14.7 | 7.1 | |
| Pomponi (2016) [62] | Italy, N=28 | 42.7 | 68.6 (6.7) | | 53.6 | 100 | 0 |  | 69.5 (5.1) | | 46.4 | 8.4 | |
| Juneja (2020) [63] | India, N=47.41 | 47.4 | 52.3 (6.8) | | 72.0 | 76.0 | 24.0 |  | 61.5 (6.7) | | 32.0 | n/a | |
| *ZBI = Zarit Burden Inventory | |  |  |  | |  |  |  |  |  | | |  |

| **Table 2: Characteristics of informal caregivers and people with PD** | |  |
| --- | --- | --- |
|  | Caregivers of people with PD (N=504) | People with PD  (N=504) |
| **Age**, mean + SD | 67.6 + 9.2 | 69.9 + 8.1 |
| **Women**, n (%) | 337 (66.9) | 183 (36.3) |
| **Education**, n (%) |  |  |
| Primary education | 15 (3.0) | 20 (4.0) |
| Secondary education | 139 (27.6) | 130 (25.9) |
| Higher education | 350 (69.4) | 352 (70.1) |
| **Marital status**, n (%) |  |  |
| Married | 446 (88.5) | 434 (86.1) |
| Living with partner | 41 (8.1) | 38 (7.5) |
| Divorced | 4 (0.8) | 3 (0.6) |
| Widow/widower | 1 (0.2) | 15 (3.0) |
| Single/unmarried | 8 (1.6) | 10 (2.0) |
| **Working status**, n (%) |  |  |
| Fulltime employment | 42 (8.33) | 14 (2.8) |
| Part-time employment | 64 (12.7) | 26 (5.2) |
| Self-employed | 34 (6.7) | 17 (3.4) |
| Unemployed | 14 (2.8) | 3 (0.6) |
| Retired | 332 (65.9) | 366 (72.6) |
| Incapacitated for work | 15 (3.0) | 71 (14.1) |
| Other | 122 (24.2) | 22 (4.4) |
| **Relationship to people with PD**, n (%) |  |  |
| Partner | 457 (90.7) | n/a |
| Child | 21 (4.2) | n/a |
| Sister or brother | 6 (1.2) | n/a |
| Friend | 8 (1.6) | n/a |
| Other | 12 (2.4) | n/a |
| **Living situation**, n (%) |  |  |
| Living on my own | 18 (3.6) | 32 (6.3) |
| Living with my partner | 437 (86.7) | 432 (85.7) |
| Living with my partner and children | 46 (9.1) | 36 (7.1) |
| Living in an institution, i.e. nursing home | - | 2 (0.4) |
| Living independently, but receive ambulatory support | n/a | 1 (0.2) |
| **Years of caregiving,** n (%) |  |  |
| Less than a year | 61 (12.4) | n/a |
| 1 to 5 years | 235 (47.9) | n/a |
| 5 to 10 years | 89 (18.1) | n/a |
| More than 10 years | 42 (8.6) | n/a |
| I don’t provide care (yet) | 64 (13.0) | n/a |
| **Caregiving involvement**, n (%) |  |  |
| Day and night | 129 (25.6) | n/a |
| During the day | 146 (30.0) | n/a |
| 3 to 6 times per week | 12 (2.4) | n/a |
| 1 to 2 times per week | 19 (3.8) | n/a |
| Less than once per week | 32 (6.3) | n/a |
| Less than once per month | 52 (10.3) | n/a |
| Very variable | 114 (22.6) | n/a |
| **Caregiver burden,** mean + SD | 15.9 + 11.7 | n/a |
| Little or no burden (0 - 20) | 366 (72.6) | n/a |
| Mild to moderate burden (21 - 40) | 118 (23.4) | n/a |
| Moderate to severe burden (41 - 60) | 20 (4.0) | n/a |
| Severe burden (61 – 88) | - | n/a |
| **Type of diagnosis**, n (%) |  |  |
| Parkinson’s Disease | n/a | 485 (96.2) |
| Atypical Parkinsonism | n/a | 19 (3.8) |
| **Time since diagnosis**, mean + SD | n/a | 7.2 + 5.2 |
| *PD: Parkinson’s Disease; n/a: not applicable* | | |

| **Table 3: Exploratory factor analysis** | | | | | |
| --- | --- | --- | --- | --- | --- |
| Factors of ZBI | Item loading | Communality^*^ | Eigen-value^**^ | Percent of variance | α-value |
| *Factor 1: Role intensity & resource strain* |  |  | 2.97 | 40.3% | 0.90 |
| 1. Patient asking for too much help | 0.384 | 0.21 |  |  |  |
| 2. Not enough time for caregiver | 0.791 | 0.53 |  |  |  |
| 3. Worrying about fulfilling different responsibilities | 0.597 | 0.52 |  |  |  |
| 8. Patient is dependent on caregiver | 0.691 | 0.46 |  |  |  |
| 10. Health affected | 0.503 | 0.44 |  |  |  |
| 12. Social life suffering | 0.650 | 0.52 |  |  |  |
| 14. Expected to be the only carer | 0.635 | 0.41 |  |  |  |
| 16. Feel unable to take care of the patient much longer | 0.465 | 0.36 |  |  |  |
| 17. Sense of losing control over life | 0.580 | 0.55 |  |  |  |
| 18. Wish somebody would take care over | 0.502 | 0.42 |  |  |  |
| 22. Feel burdened | 0.697 | 0.60 |  |  |  |
| *Factor 2: Social restrictions & anger* |  |  | 1.24 | 7.0% | 0.79 |
| 4. Embarrassed about patient’s behavior | 0.608 | 0.27 |  |  |  |
| 5. Feel angry | 0.543 | 0.35 |  |  |  |
| 6. Negative effects on other relationships | 0.496 | 0.26 |  |  |  |
| 9. Feel strained | 0.623 | 0.44 |  |  |  |
| 13. Feeling uncomfortable having friends visit because of patient | 0.600 |  |  |  |  |
| *Factor 3: Self-criticism* |  |  | 1.13 | 5.9% | 0.76 |
| 20. Feel should be doing more | 0.818 | 0.17 |  |  |  |
| 21. Feel could do a better job | 0.682 | 0.24 |  |  |  |
| *Only loadings ≥ .35 are shown. ZBI: Zarit Burden Interview.; ^*^Communality =*  the total amount of variance that can be explained by a given principal component; ^**^Eigenvalue= the proportion of each variable's variance that can be explained by the factors | | | | | |

| **Table 4: Characteristics of caregivers and people with PD as predictors for caregiver burden** | | | | | |  |  |
| --- | --- | --- | --- | --- | --- | --- | --- |
| Predictors | Model I | | Model II | | Model III | |  |
|  | B | 95% CI | B | 95% CI | B | 95% CI |  |
| ***Caregiver assessments*** |  |  |  |  |  |  |  |
| *Age* |  |  |  |  |  |  |  |
| <50 years | ref. | ref. |  |  | ref. | ref. |  |
| 50 – 70 years | 0.067 | -0.28 – 0.42 |  |  | 0.162 | -0.20 – 0.52 |  |
| > 70 years | 0.113 | -0.26 – 0.49 |  |  | 0.160 | -0.23 – 0.55 |  |
| Women | 0.057 | -0.07 – 0.19 |  |  | -0.014 | -0.16 – 0.13 |  |
| *Marital status* |  |  |  |  |  |  |  |
| Married | - 0.001 | -0.22 – 0.21 |  |  | 0.040 | -0.18 – 0.26 |  |
| Unmarried | ref. | ref. |  |  | ref. | ref. |  |
| *Educational level* |  |  |  |  |  |  |  |
| Primary education | - 0.032 | -0.39 – 0.33 |  |  | -0.064 | -0.45 – 0.32 |  |
| Secondary education | - 0.036 | -0.39 – 0.32 |  |  | -0.099 | -0.48 – 0.28 |  |
| Tertiary education | ref. | ref. |  |  | ref. | ref. |  |
| *Work status* |  |  |  |  |  |  |  |
| Working | 0.006 | -0.16 – 0.17 |  |  | 0.060 | -0.11 – 0.23 |  |
| Not working | ref. | ref. |  |  | ref. | ref. |  |
| *Caregiver involvement* |  |  |  |  |  |  |  |
| Day and night | ref. | ref. |  |  | ref. | ref. |  |
| During the day | - 0.006 | -0.17 – 0.16 |  |  | -0.013 | -0.18 – 0.16 |  |
| 3 to 6 times per week | 0.200 | -0.23 – 0.63 |  |  | 0.225 | -0.21 – 0.66 |  |
| 1 to 2 times per week | - 0.232 | -0.59 – 0.13 |  |  | -0.219 | -0.59 – 0.16 |  |
| Less than once per week | - 0.232 | -0.51 – 0.04 |  |  | -0.235 | -0.52 – 0.05 |  |
| Less than once per month | - 0.085 | -0.31 – 0.14 |  |  | -0.025 | -0.28 – 0.23 |  |
| Very variable | 0.05 | -0.13 – 0.23 |  |  | 0.050 | -0.14 – 0.24 |  |
| Caregiver activation | - 0.006 | -0.02 – 0.01 |  |  | -0.006 | -0.03 – 0.01 |  |
| Perceived social support | - 0.116*** | -0.17 – - 0.07 |  |  | -0.108*** | -0.16 – -0.06 |  |
| *Coping strategies* |  |  |  |  |  |  |  |
| Problem-focused coping | 0.346*** | 0.20 – 0.49 |  |  | 0.352*** | 0.21 – 0.50 |  |
| Emotion focus coping | 0.200 | -0.13 – 0.37 |  |  | 0.081 | -0.18 – 0.34 |  |
| Avoidant coping | 0.947*** | 0.71 – 1.19 |  |  | 0.973*** | 0.73 – 1.21 |  |
| **People with PD assessments** |  |  |  |  |  |  |  |
| *Age* |  |  |  |  |  |  |  |
| <50 years |  |  | ref. | ref. | - | - |  |
| 50 – 70 years |  |  | 0.108 | -0.72 – 0.93 | - | - |  |
| > 70 years |  |  | 0.086 | -0.74 – 0.91 | - | - |  |
| Women |  |  | - 0.003 | -0.17 – 0.16 | - | - |  |
| *Educational level* |  |  |  |  |  |  |  |
| Primary education |  |  | ref. | ref. | ref. | ref. |  |
| Secondary education |  |  | - 0.140 | -0.53 – 0.25 | -0.307 | -0.64 – 0.03 |  |
| Tertiary education |  |  | -0.206 | -0.59 – 0.18 | -0.271 | -0.60 – 0.06 |  |
| *Work status* |  |  |  |  |  |  |  |
| Working |  |  | - 0.300 | -0.48 – 0.02 | -0.254* | -0.47 – -0.04 |  |
| Not working |  |  | ref. | ref. | ref. | ref. |  |
| *Coping strategies* |  |  |  |  |  |  |  |
| Taking action |  |  | 0.077 | -0.10 – 0.26 | 0.066 | -0.09 – 0.22 |  |
| Distancing |  |  | - 0.117 | -0.27 – 0.03 | -0.072 | -0.20 – 0.06 |  |
| Goal oriented |  |  | - 0.040 | -0.20 – 0.12 | -0.012 | -0.15 – 0.13 |  |
| Social support |  |  | 0.048 | -0.07 – 0.17 | 0.004 | -0.10 – 0.10 |  |
| Avoidance |  |  | 0.056 | -0.12 – 0.23 | 0.071 | -0.09 – 0.22 |  |
| Disease duration |  |  | 0.004 | -0.1 – 0.02 | 0.001 | -0.01 – 0.01 |  |
| Depression |  |  | 0.009 | -0.01 – 0.02 | 0.002 | -0.01 – 0.01 |  |
| Anxiety |  |  | 0.005 | -0.01 – 0.02 | 0.002 | -0.01 – 0.02 |  |
| Motor symptoms |  |  | 0.145 | -0.10 – 0.39 | 0.251* | 0.04 – 0.46 |  |
| Activities of daily living |  |  | - 0.009 | -0.02 – 0.00 | -0.011* | -0.02 – 0.00 |  |
| *** = < .001, ** = < .01, * < .05; Model 1: regression analysis with only caregiver characteristics as predictors; Model 2: regression analysis model with only characteristics of people with PD; Model 3: regression analysis model with both characteristics of caregivers and people with PD: age and gender variables of people with PD were excluded due to severe collinearity with other predictors | | | | | | | |

| **Table 5: Multivariate linear regression analysis on total ZBI and subscales*** | | |  |
| --- | --- | --- | --- |
| Dependent variable and significantly associated variables. | Coefficient | p-value | 95% CI |
| ***Dependent variable: Total ZBI score*** |  |  |  |
| Social support | -0.108 | <.001 | -0.16 – -0.06 |
| Problem-focused coping | 0.352 | <.001 | 0.21 – 0.50 |
| Avoidant coping | 0.973 | <.001 | 0.73 – 1.21 |
| Working* | -0.254 | <.05 | -0.47 – -0.04 |
| Motor symptoms* | 0.251 | <.05 | 0.04 – 0.46 |
| Activities of daily living* | -0.011 | <.05 | -0.02 – 0.00 |
| ***Dependent variable: Role intensity & resource strain*** |  |  |  |
| Problem-focused coping | 0.249 | <.001 | 0.13 – 0.37 |
| Avoidant coping | 0.759 | <.001 | 0.56 – 0.96 |
| Social Support | -0.066 | <.01 | -0.11 – -0.02 |
| Motor symptoms* | -0.171 | <.05 | -0.00 – 0.34 |
| ***Dependent variable: Social restriction and anger*** |  |  |  |
| Avoidant coping | 0.770 | <.001 | 0.60 – 0.94 |
| ***Dependent variable: Self-criticism*** |  |  |  |
| Problem-focused coping | 0.347 | <.001 | 0.20 – 0.49 |
| Avoidant coping | 0.624 | <.001 | 0.38 – 0.87 |
| Caregiver activation | -0.018 | <.01 | -0.03 - -0.01 |
| Social support | -0.058 | <.05 | -0.11 – 0.01 |
| Avoidant coping* | 0.166 | <.05 | 0.01 – 0.32 |
| Motor symptoms* | 0.232 | <.05 | 0.02 – 0.45 |
| Activities of daily living* | -0.013 | <.01 | -0.02 - -0.00 |
| *only significant results are displayed in this table; **characteristic of people with PD;  ZBI = Zarit Burden Interview | | |  |
|  |  |  |  |
|  |  |  |  |
|  |  |  |  |

|  | | **Table 6: Characteristics of subsample of participants in interview study** | | | | | | | | |
| --- | --- | --- | --- | --- | --- | --- | --- | --- | --- | --- |
| Nr. | Relationship | | Disease type | Sex | Age | Working status | Time since PD diagnosis | Duration caregiving involvement | Caregiver Burden (ZBI*) | |
|  |  |  |  |  |  |  |  |  | *Score* | *Category* |
| C01 | Partner | | PD | Woman | 75 | Retired | 8 years | >5 years | 48 | Moderate to severe burden |
| C02 | Partner | | PD | Man | 83 | Retired | 2 years | 2-5 years | 17 | Little or no burden |
| C03 | Partner | | PD | Man | 70 | Retired | 1 year | <1 year | 9 | Little or no burden |
| C04 | Partner | | PD | Man | 59 | Parttime | 6 years | 2 - 5 years | 30 | Mild to moderate burden |
| C05 | Partner | | PD | Woman | 65 | Retired | 6 years | >5 years | 11 | Little or no burden |
| C06 | Partner | | PD | Woman | 57 | Fulltime | 2 years | 1 year | 6 | Little or no burden |
| C07 | Partner | | PD | Woman | 58 | Parttime | 9 years | >5 years | 43 | Moderate to severe burden |
| C08 | Partner | | Atypical parkinsonism | Woman | 73 | Retired | 3 years | <1 year | 13 | Little or no burden |
| C09 | Daughter** | | PD | Woman | 50 | Parttime | 2 years | 2-5 years | 34 | Mild to moderate burden |
| C10 | Son | | PD | Man | 53 | Unemployed | 2 years | <2 years | 13 | Little or no burden |
| C11 | Partner | | PD | Woman | 47 | Fulltime | 5 years | 2-5 years | 19 | Little or no burden |
| C12 | Daughter | | PD | Woman | 51 | Parttime | 14 years | 2-5 years | 42 | Moderate to severe burden |
| C13 | Partner | | PD | Woman | 74 | Retired | 20 years | NA*** | 40 | Mild to moderate burden |
| C14 | Partner | | PD | Woman | 77 | Retired | 4 years | 2-5 years | 31 | Mild to moderate burden |
| C15 | Son | | PD | Man | 47 | Fulltime | 8 years | 1-2 years | 5 | Little or no burden |
| C16 | Friend | | PD | Woman | 62 | Fulltime | 9 years | 2-5 years | 15 | Little or no burden |
| C17 | Partner | | PD | Woman | 71 | Retired | 7 years | NA | 11 | Little or no burden |
|  | | *ZBI =Zarit Burden Interview; ** at the time of the interview the mother with PD was deceased, *** NA=missing data | | | | | | | | |

| **Table 7: Main themes of qualitative analysis and illustrative quotes *** | | | |
| --- | --- | --- | --- |
| Themes | | | Illustrative quotes |
| 1 | Dealing with signs and symptoms of cognitive decline | | “I think that cognitive decline is the worst. You cannot really have a conversation anymore.” [C07].  “He is actually a big child that you have to take by the hand so that he takes his pills, that he gets dressed, that he goes to the dentist on time.” [C08]  “What has become the heaviest challenge is that he has become quite forgetful in recent years. And you know that he cannot do anything about it, it’s the stupid Mr. Parkinson that bothers him, but it can be a huge problem from time to time. Especially, when I am tired myself. Then I sometimes freak out: ‘oh, you have already asked that five times!’ You know that this is not right, it gives you a send of failure that you were that dismissive.” [C14] |
| 2 | Psychological and emotional well-being | | “I can be cheerful, but my heart is crying. I can easily switch. I can enjoy so many beautiful things, but my life has changes so much that I am always sad. Always. […] I am not jealous of other people. I wish others to be happy. At the same time, I recently looked up old photos from the time we were together […] where we were sitting together under a palm tree and his hands around me. Then I really crave for this memories. [PD] did something to my mental health. The happiness in my life is gone.” [C01] |
| 3 | Impact on everyday life | | If you live with someone with Parkinson’s Disease, your life is very structured. You cannot longer do something spontaneously. This has all to do with the strict times in which the medications need to be taken. […] I’m fine with it. But it does mean that you live by the hour. By the clock.” [C04]  “If you need to stay at home more often, you cancel more things. Things he couldn’t or he didn’t want to. Or if we were going somewhere and he wanted to leave after 10 minutes. Then your social contacts become less.” [C13]. |
| 4 | Impact on the relationship | Subtheme: Relationship with the person with PD  Subtheme: Relationship with others | “In the beginning I challenge him. I went beyond my limits by for example cleaning the gutters, so that he would say ‘watch out!’, but he stopped saying it. He is not understanding anymore. He is not seeing it anymore” [C01]  “He was a biker. He cycled to Hungary, to Italy, around the Ijsselmeer. He was a very strong and powerful man. When we went bicycling together and we had to wait at a traffic light, he would always say: ‘You’ll get an ice cream soon” and then he would push me like a father would push a child. He would put his hand on my back and push me so that we can sit on a patio immediately. Now it’s completely the other way around. He pushed me, but now I must be in front. I must carry him on my shoulders. That’s now our relationship on all domains. I carry him.” [C01]  “He has freezing problems and he falls quite often. But if you see him, you think that it is going well. You do not see that he has Parkinson’s disease. And because of the lack of visible symptoms, many outsiders get a wrong idea of the real impact.” [C13]  “When people say ‘if I can ever do something for you’, I do not know what to answer. When someone says to me: “come sit down for a while, I’ll take it over from here’, then it really takes the load off my shoulders. It might be a small difference in nuance, but it does have a big impact […] I really need the initiative from my environment.” [C01] |
| 5 | Concerns about the future | | “What I find very difficult are the fears that she has about the future. It is not an everyday topic, but it becomes evident now and then. As a young woman she has worked in a nursing home with Parkinson’s patients, and she still has that image of how people then used to be: hanging in a chair or just lying in bed. And she is afraid of that. She can be terribly sad about that […] and I have to deal with her fears.” [C04] |
| 6 | Positive impact | | “Parkinson taught me to slow down. I never again will say ‘I need to quickly do this’ I do not do two things at the same time, never do anything spontaneously and there are no surprises anymore” [C02]  “We are doing it together.” [C11] |
| 7 | Coping mechanisms | | “When I meditate, I cry. Very often. Then I cry and all is gone.” [C01] |
| *Quotes are translated from Dutch to English | | | |

**Table 8: Merged data from quantitative and qualitative research findings**

| Topic | Quantitative findings | Qualitative findings | Integrated findings |
| --- | --- | --- | --- |
| Relationships and social support | Informal caregivers that reported more perceived social support, reported a lower perceived caregiver burden. | Informal caregivers experienced several challenges regarding the *relationship with the person with PD* that impacted the perceived caregiver burden. Several informal caregivers reported role-reversal, sense of loss of the relationship, feeling disconnected from person with PD, and becoming more and more a carer than a partner. By contrast, others reported that PD has deepened the relationship with the person with PD and increased mutual understanding.  Informal caregivers also reported that PD also impacted their *relationship with others.* They felt confronted with incomprehension from their surroundings and also felt a lack of flexibility and independence to continue with their social life. | **Expansion:**  PD impacts the relationship between the informal caregiver and the person with PD, which can have negative as well as positive impact on perceived caregiver burden.  **Confirmation:**  Social support has a positive impact on perceived caregiver burden. |
| Coping strategies | Problem-focus and avoidant coping behavior was associated with a higher perceived caregiver burden. | Informal caregivers reported the use of emotional coping as well as problem-focused to be prepared for the future. | **Discordance:**  Problem-focused coping can have a positive as well as negative impact on perceived caregiver burden. |
| Motor and non-motor symptoms | Informal caregivers reporting that the person with PD had higher motor impairments and higher impairments in activities of daily living, had a higher perceived caregiver burden. | Informal caregivers reported that the burden of dealing with signs and symptoms of cognitive decline outsight the impact of motor symptoms. Cognitive decline put extra burden on informal caregivers through a higher dependence of the person with PD on support from the informal caregiver, including performing additional tasks. In addition, it was related to the the fear of losing the person they were used to know, and the forgetfulness may lead to negative feelings such as sadness, anger and frustration. | **Expansion:**  Severity of motor symptoms and the related impairment of performing activities of daily living negatively impact perceived caregiver burden.  Informal caregivers reported that signs and symptoms of cognitive decline have a more severe impact on perceived caregiver burden. |
| Concerns about the future | - | Informal caregiver indicated that they were confronted with both: (1) Fear and worries of the person with of PD, related to disease progression and severity of symptoms; and (2) own fears and worries about the future way of living and dealing with the impact of PD. | **Expansion:**  Concerns of the impact of PD on one’s own future as well as on the disease progression and coping of the person with PD negatively impacts perceived caregiver burden. |
| Impact on everyday life | - | Informal caregivers reported on perceived restrictions and limitations due to PD, which included lack of freedom, lack of autonomy due to a change in daily routines. | **Expansion:**  Lack of freedom and autonomy of informal caregivers negatively impacts perceived caregiver burden. |
| Psychological and emotional well-being | No association was found for impact of feelings of anxiety and symptoms of depression of the person with PD on perceived caregiver burden. | Informal caregivers worried about the disease progression, as well as the concern to not be able to leave the person with PD home alone. In addition, informal caregivers reported on feeling of sadness as they experience a sense of losing the relationship, feeling to live a different life, and the need to give up social and family life. Moreover, informal caregivers reported feeling guilty when they felt unable to deal with cognitive and behavioral impairments. | **Expansion:**  Informal caregivers’ emotion and psychological well-being impacts perceived caregiver burden. |
| Positive impact of PD | - | Informal caregivers reported that PD also had a positive impact on their life and relationship. They experienced more calmness due to living a more structured life, and a stronger relationship with the person with PD | **Expansion:**  PD has not only a negative impact on the life and relationship of informal caregivers, which positively impacts the caregiver burden. |
