# Supplementary material for: Caregiver burden in Parkinson’s disease: a mixed-methods study
Source: BMC Med. 2023 Jul 10;21:247. doi: 10.1186/s12916-023-02933-4 (PMC10332089; doi:10.1186/s12916-023-02933-4)
Supplement: Supplementary file 3 — Additional file 3: Supplemental file 1. PubMed search strategy. Supplemental file 2. Translated interview guide. Supplemental file 3. Code tree. [file 12916_2023_2933_MOESM3_ESM.docx]

**SUPPLEMENTAL DATA I**

PubMed Search Strategy

(((“Parkinson Disease”[MeSH Major Topic] OR Parkinson*[Title/Abstract]) AND (Caregiver[Title/Abstract] OR carer[Title/Abstract]) AND (ZARIT[Title/Abstract] OR ZBI[Title/Abstract] OR Burden[Title/Abstract]))

**SUPPLEMENTAL DATA II**

**Translated Interviewguide**

**Main questions:**

What is the impact of Parkinson’s Disease on the lives of those caring for a person with Parkinson’s Disuse? In what way do they experience caregiver burden?

**Introduction/Background**

- Tell me something about yourself.
- About your family and work situation
- What is your relationship to the person with Parkinson’s Disease?
- Are you the only person taking care? Are other people as well involved (think about family, friends, neighbours, colleagues, etc)

**Experience**

- Can you tell me something about your experience in caring for a person with PD?
- How does it affect your life?

**Diagnosis**

- How did the process of diagnosing Parkinson’s Disease go?
- Hoe did you experience this progress?
- How was this message perceived?
- What were you worried about?
- What did you need at this moment?

**Current load**

- What kind of caregiving tasks do you perform?
- How does performing these tasks affect your life?
- To what extend do you feel burdened? Why/why not?
- What difficulties do you experience?
- Do you have mental or physical complaints as result of providing informal care?
- Has caring changed you? Your bevor, how you approach your life?
- What positive or negative aspects do you associated with caring for a person with Parkinson’s Disease?

**Relationship**

- Does the disease affect your relationship? How/In what way?
- How is the interaction/communication? Have things changed?
- Has the division of roles changed?
- In what way would you say that there is mutual understanding for the situation?
- What are your needs regarding your relationship? Are your needs met? Why/Why not?
- Does the disease affect your own social life? How and why?
- How do you deal with these changes?
- Does the environment influence how you perceived the disease? How?

**Physical and mental symptoms/Well-being**

- How doe symptoms associated with Parkinson’s Disease affect your life?
- Which symptoms are the most difficult to deal with for you, your partner and your life? Why?

**Future perspective**

- When you think about the future, what comes to your mind?
- How does the disease affect your future/expectations?
- What are you most afraid of/most worried about?

**Closing**

- Do you have any other comments?
- Are there any other aspects that have not been covered so far that you would like to share?

**** **SUPPLEMENTAL DATA III**

**Code tre**
